# Development of the Multiple Gene Knockout System with One-Step PCR in Thermoacidophilic Crenarchaeon *Sulfolobus acidocaldarius*

**DOI:** 10.1155/2017/7459310

**Published:** 2017-10-31

**Authors:** Shoji Suzuki, Norio Kurosawa

**Affiliations:** Department of Science and Engineering for Sustainable Development, Faculty of Science and Engineering, Soka University, 1-236 Tangi-machi, Hachioji, Tokyo 192-8577, Japan

## Abstract

Multiple gene knockout systems developed in the thermoacidophilic crenarchaeon *Sulfolobus acidocaldarius* are powerful genetic tools. However, plasmid construction typically requires several steps. Alternatively, PCR tailing for high-throughput gene disruption was also developed in *S. acidocaldarius*, but repeated gene knockout based on PCR tailing has been limited due to lack of a genetic marker system. In this study, we demonstrated efficient homologous recombination frequency (2.8 × 10^4^ ± 6.9 × 10^3^ colonies/*μ*g DNA) by optimizing the transformation conditions. This optimized protocol allowed to develop reliable gene knockout via double crossover using short homologous arms and to establish the multiple gene knockout system with one-step PCR (MONSTER). In the MONSTER, a multiple gene knockout cassette was simply and rapidly constructed by one-step PCR without plasmid construction, and the PCR product can be immediately used for target gene deletion. As an example of the applications of this strategy, we successfully made a DNA photolyase- (*phr-*) and arginine decarboxylase- (*argD-*) deficient strain of *S. acidocaldarius*. In addition, an agmatine selection system consisting of an agmatine-auxotrophic strain and *argD* marker was also established. The MONSTER provides an alternative strategy that enables the very simple construction of multiple gene knockout cassettes for genetic studies in *S. acidocaldarius*.

## 1. Introduction

High-throughput PCR tailing for gene disruption has been developed in the thermoacidophilic crenarchaeon *Sulfolobus acidocaldarius* [1]. We attempted to improve this technique and develop an efficient multiple gene knockout strategy with a PCR tailing (one-step PCR) method.

Gene knockout via homologous recombination is a powerful tool for the generation of specific mutants and subsequent functional analysis of the gene. Three unmarked gene deletion methodologies, that is, plasmid integration and segregation (PIS), marker replacement and looping out (MRL), and marker insertion and unmarked target gene deletion (MID), have been employed in *S. acidocaldarius* and *S. islandicus* [2–4]. These pop-out recombination-based approaches are effective for multiple gene knockout [5–7], but plasmid construction is required. In contrast, one-step PCR followed by a marker replacement system using the *pyrE* selection marker flanked by 40–50 bp of homologous regions, for example, 5′ and 3′ flanking regions of the target gene, has been developed in *S. acidocaldarius* [1]. This PCR-tailing method allows for effective, high-throughput gene functional analysis without plasmid construction [1]. However, this method was not sufficient for repeated gene disruptions because only the uracil selection system (pyrimidine-auxotrophic strain and selectable marker [*pyrE*] gene) was available in *S. acidocaldarius*. A pop-out recombination system using one-step PCR for multiple gene knockout has not been reported in hyperthermophilic archaea. Furthermore, the homologous recombination efficiency using the PCR-tailing technique has not been reported [1].

We recently constructed the restriction endonuclease *Sua*I-deficient *S. acidocaldarius* strain SK-1 (Δ*pyrE* Δ*suaI*), which has the potential for efficient and flexible direct modification of the genome using synthetic oligonucleotides or PCR products without any methylation procedures [8]. In our current study, we estimated the effects of transformation conditions (plating methods, DNA topology, CaCl_2_ treatment, recovery buffer, growth phase of cells, DNA volume, and flanking region length) on homologous recombination efficiency and optimized the transformation protocol for PCR tailing. If a combination system consisting of one-step PCR and pop-out excision is developed, alternative multiple gene knockout systems become accessible. To this end, *pyrE-lacS* dual marker genes were utilized for positive, negative, and blue selection. This effective approach (multiple gene knockout system with one-step PCR) was validated by unmarked gene knockout of the DNA photolyase- and arginine decarboxylase-encoding genes (*phr* and *argD*, resp.) in the *Sua*I-deficient *S. acidocaldarius* strain SK-1 (Δ*pyrE* Δ*suaI*).

## 2. Materials and Methods

### 2.1. Strains and Growth Conditions

The strains used in this study are listed in Table 1. The *S. acidocaldarius* pyrimidine-auxotrophic and restriction endonuclease *Sua*I-deficient strain SK-1 (Δ*pyrE* Δ*suaI*) was used as basic host strain [8]. This strain and its derivatives were cultivated in xyrose and tryptone (XT) medium (pH 3) [9] containing 1× basal salts (3 g K_2_SO_4_, 2 g NaH_2_PO_4_, 0.3 g MgSO_4_·7 H_2_O, and 0.1 g CaCl_2_·2H_2_O), 20 *μ*L of trace mineral solution (1 mg FeCl_3_·6H_2_O, 0.1 mg CuCl_2_·2 H_2_O, 0.12 mg CoSO_4_·7 H_2_O, 0.1 mg MnCl_2_·4 H_2_O, and 0.1 mg ZnCl_2_), 2 g/L xyrose, and 1 g/L tryptone in 1 L Milli-Q H_2_O at 75°C with or without shaking (160 rpm). To solidify plates, identical components of 1× basal salts containing 2.9 g MgSO_4_·7 H_2_O and 0.5 g CaCl_2_·2 H_2_O were used. For growth of the uracil-auxotrophic strain, 0.02 g/L uracil was added to XT medium (XTU). XTU medium supplemented with 50 *μ*g/mL 5-FOA was used for counterselection with the pop-out recombination method. For cultivation of the *argD* mutant, 1 mg/mL agmatine (agmatine sulfate [Tokyo Chemical Industry]) was added to the XTU medium. *Escherichia coli* strain DH5*α*, used for general manipulation, was routinely cultivated at 37°C in Luria–Bertani medium supplemented with ampicillin (100 *μ*g/mL).

### 2.2. General DNA Manipulation

The reagents used in these experiments were prepared as previously described [8]. PCR products and plasmid DNA were purified using NucleoSpin Gel and PCR Clean-up and NucleoSpin QuickPure kits (Macherey-Nagel), respectively.

### 2.3. Construction of Marker Cassettes

The plasmid and linear DNA used in this study are shown in Table 1 and the PCR primers used are listed in Table 2.

#### 2.3.1. Construction of Marker Cassettes for Estimation of Homologous Recombination Efficiency

**Figure 1:**
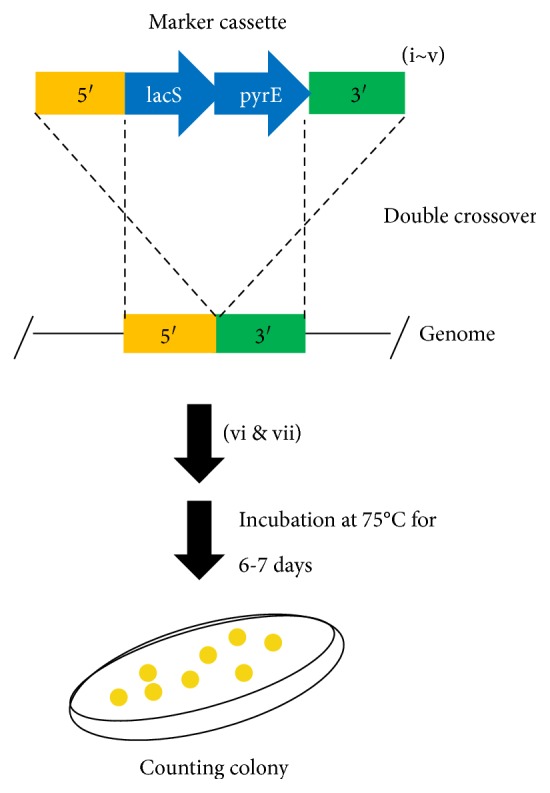
Schematic and optimization of transformation procedure. A marker cassette containing the 5′ and 3′ homologous regions of the target locus at both ends of *pyrE*-*lacS* marker genes was electroporated into strain SK-1 (Δ*pyrE* Δ*suaI*) under various conditions: (i) DNA topology, (ii) CaCl_2_ treatment, (iii) growth phase of competent cells, (iv) DNA volume, and (v) length of flanking region. After electroporation, cells were cultivated in recovery buffer (vi) as needed and plated onto XT plates by spreading or overlay plating (vii). The resulting colonies were defined as transformants and counted.

We constructed the plasmid placSpyrE, which contains marker cassettes of approximately 800 bp of the 5′ and 3′ homologous regions of the *suaI* (Saci_1976) locus at both ends of the *pyrE*-*lacS* marker. The *lacS* gene, together with its putative promoter and terminator regions, was amplified from the *S. solfataricus* P2 genomic DNA using primers SSOlacS-F/R (containing PstI/BamHI restriction sites). The PCR product was digested with PstI/BamHI, then purified and inserted into pSuaIPOP [8] at the corresponding restriction sites. Linear DNA of the *pyrE*-*lacS* dual marker cassette containing various lengths (800, 50, 40, 30, 20, and 10 bp) of the 5′ and 3′ homologous arms was amplified from placSpyrE as a template using the corresponding primers (E800-20-F/R and E10-2F/2R) and Emerald Amp MAX PCR Master mix (Takara Bio). The PCR products were purified in 5 mM Tris-HCl (pH 8.5) and transformed into SK-1 to estimate the homologous recombination efficiency via double crossover (Figure 1).

#### 2.3.2. Construction of *phr* and *argD* Knockout PCR Products

A MID strategy [3] and PCR-tailing technique [1] were combined to develop our multiple gene knockout system with one-step PCR (MONSTER). The MONSTER was utilized for *phr* (Saci_1227) and *argD* (Saci_1363) knockout cassette construction. In brief, the *phr* knockout PCR product (MONSTER-phr) was amplified from placSpyrE as a template using primers phr-pop-F/R (containing the 48 bp and 30 bp 5′ and 3′ flanking regions of *phr* and a 48 bp region of *phr* as the Tg-arm) and Premix Taq (Ex Taq Version 2.0; Takara Bio) under the following conditions: 94°C for 3 min; 30 cycles of 94°C for 30 s, 56°C for 30 s, and 72°C for 3 min, and a final extension for 3 min. Similarly, the *argD* knockout PCR product (MONSTER-argD) was amplified from placSpyrE as a template using primers argD-pop-F/R (containing a 48 bp region of *argD* as the Tg-arm and the 30 bp and 48 bp 5′ and 3′ flanking regions of *argD*) and the *LA-Taq* DNA polymerase (Takara Bio) under same PCR conditions. The purified PCR products were used in subsequent experiments.

#### 2.3.3. Construction of an *argD*-Based Shuttle Vector

The *S. solfataricus argD* gene with approximately 100 bp of the 5′ and 3′ flanking regions was amplified by PCR using the primers SsoargD-KpnI-F/PstI-R, which contain the KpnI and PstI restriction sites, respectively, and Premix Taq (Ex Taq Version 2.0; Takara Bio). The *SsopyrEF* marker genes in pSAV2 [8] were replaced by the *SsoargD* marker gene at the KpnI and PstI sites, thus generating the *argD*-based shuttle vector pSAV2-argD.

### 2.4. Transformation Procedure

Preparation of electrocompetent cells and transformation were completed as previously described [8] with the following modifications. Cells were incubated in a 1 L DURAN bottle (Schott) containing 200 mL of medium with shaking using a Bio shaker (TAITEC). *S. acidocaldarius* (strain SK-1 [Δ*pyrE* Δ*suaI*]) electrocompetent cells for transformation with a shuttle vector and via homologous recombination were prepared from a late log to stationary phase culture (OD_600_ ≧ 0.7) and an early to midlog phase culture (OD_600_ = 0.1–0.4) incubated in XTU medium, respectively. Cells were harvested by centrifugation (10160 ×g for 15 min at 25°C) using a Kubota 6500 and were washed once in 0.3 volumes of the original culture volume of 20 mM sucrose at room temperature. The final optical density at 600 nm (OD_600_) of cells was adjusted to 5.9 (2 × 10^9^ cells/mL) by calculation, and aliquots were frozen at −84°C in an ultralow freezer (Sanyo). All transformation procedures (including preparation of competent cells) were carried out at room temperature. Two hundred microliters of competent cells (4 × 10^8^ cells) were thawed by hand and mixed with 1–10 *μ*L of DNA in 5 mM Tris-HCl (pH 8.5). For the CaCl_2_ treatment, 40 mM CaCl_2_ was added to cells at a final concentration of 0.1–0.4 mM CaCl_2_. After pipetting or vortexing, approximately 200 *μ*L of the competent cell-DNA mixture was transferred to a 2 mm electroporation cuvette (Bio-Rad or NeppaGene). Electroporation was performed using the Gene Pulser II (Bio-Rad) set to a 2.5 or 3.0 kV exponential decay pulse form for 9 or 20 ms, respectively. After electroporation, regeneration was performed as needed. *Sulfolobus* cells were immediately transferred into 800 *μ*L of recovery buffer consisting of 20 mM sucrose; 2× basal4 (modified 2× basal salts with 5.75 g MgSO_4_·7 H_2_O and 1 g CaCl_2_·2 H_2_O, 40 *μ*L of trace mineral solution, and 50 *μ*L of 50% H_2_SO_4_ in 1 L of Milli-Q H_2_O); a previously described incubation solution (0.3% (NH_4_)_2_SO_4_, 0.05% K_2_SO_4_, 0.01% KCl, and 0.07% glycine, pH 4.7) [10] with a modified pH (named Buffer C in this study); and modified Brock's basal salt mixture (MBS), pH 4.7 (1.3 g (NH_4_)_2_SO_4_, 0.2 g KH_2_PO_4_, 0.25 g MgSO_4_·7 H_2_O, 0.07 g CaCl_2_·2 H_2_O, 2.0 mg FeCl_3_·6 H_2_O, 1.8 mg MnCl_2_·4 H_2_O, 4.5 mg Na_2_B_4_O_7_·10 H_2_O, 0.22 mg ZnSO_4_·7 H_2_O, 0.05 mg CuCl_2_·2 H_2_O, 0.03 mg Na_2_MoO_4_·2 H_2_O, 0.03 mg VOSO_4_·2 H_2_O, and 0.01 mg CoSO_4_·7 H_2_O in 1 L of Milli-Q H_2_O) [11]. Cells were then incubated at 77°C–78°C for 30 min without shaking in a hot block (TOHO). After the regenerated samples were centrifuged (11000 ×g for 1 min at 25°C), 800 *μ*L of supernatant was removed and the pellet was suspended in 200 *μ*L followed by spreading on plates. Two plating methods, that is, direct plating and overlay cultivation, were performed. For direct plating, the transformed cells were immediately spread onto XT plates and incubated at 75°C for 6-7 days in sealed plastic cases. For overlay cultivation, transformed cells (~1 mL) were mixed with 10 mL of prewarmed top gel solution (5 mL of XT medium, 5 mL of 0.4% gellan gum, 50 *μ*L of 0.5 M CaCl_2_, and 50 *μ*L of 2 M MgSO_4_) at 75°C, then poured onto XT plates and cultivated at 75°C for 6-7 days in sealed plastic cases.

### 2.5. X-Gal Assay

*β*-Glycosidase activity encoded by the *lacS* gene was detected in transformant colonies by spraying a 10 mg/mL X-gal (Wako or Carbosynth) solution on the plates and incubating at 75°C for 1 day. Transformants (*lacS*^+^) convert the chemical into a strong blue substance, whereas nontransformants (wild-type *S. acidocaldarius*) do not [12].

### 2.6. Estimation of Transformation Efficiency

When *pyrE* or *argD* selectable marker was used for positive selection, colonies appearing on the plate were scored except for tiny colonies that might have been background.

### 2.7. Characterization of Mutant Strains

To characterize the phenotypes of the DNA photolyase-deficient strain DP-1 (Δ*pyrE* Δ*suaI* Δ*phr*) and *argD* deletion mutant SK-5 (Δ*pyrE* Δ*suaI* Δ*argD*), UV sensitivity and agmatine auxotrophy were examined, respectively.

To assess photoreactivation [1] in the strain DP-1, the growth properties under light and dark conditions after UV irradiation were examined. One milliliter of each overnight culture (late log to stationary phase) was poured in 90 × 15 mm plastic petri dishes (IWAKI) and irradiated with a UV lamp (UVM-57) (304 nm, 6 W) (Tech-jam) positioned 6.5 cm from the top of the dish at room temperature for 60 s (1200 J/m^2^). UV-irradiated cultures were immediately inoculated into 6 mL of XTU liquid medium to yield an initial OD_600_ of 0.005. Cells were then cultivated with shaking. For mock-treated control cultures, the same procedure was followed without UV irradiation. For dark conditions, test tubes and Bio shakers (TAITEC) were covered in foil. For light conditions, cells were cultivated under a white LED using ODS-LS16-W (Ohm Electric). Cell growth was monitored thereafter.

To compare the growth properties of strain SK-5 in the presence or absence of agmatine, overnight cultures (late log to stationary phase) were inoculated into 6 mL of XTU liquid medium supplemented with 100–1000 *μ*g/mL agmatine to yield an initial OD_600_ of 0.005. Cells were cultivated with shaking and cell growth was monitored thereafter.

Transformant genotypes were analyzed by sequencing the target region following PCR amplification using primer sets that anneal outside the flanking target gene locus.

## 3. Results

### 3.1. Effects of Transformation Conditions on Homologous Recombination Efficiency

The PCR-tailing technique for gene disruption was developed in the thermoacidophilic crenarchaeon *S. acidocaldarius* [1]; however, transformation efficiency has not been reported. Homologous region length can significantly impact transformation efficiency [13]. The efficiency of homologous recombination via double crossover using very short homologous arms (50~10 bp) is likely very low. The widely used transformation procedure for *Sulfolobus* has been reported; however, the effects of transformation conditions on transformation efficiency have not been characterized in detail as compared to those of other model systems [14–16]. To develop a reliable multiple gene knockout system using PCR tailing, we first optimized the following transformation conditions: plating methods, DNA topology, CaCl_2_ treatment, recovery buffer, growth phase of cells, DNA volume, and length of flanking regions (Figure 1).

We examined the effects of two plating methods on transformation efficiency (Figure 1, vii). We used 200 ng of linear DNA from pyrElacS800 (800 bp homologous arms Table 1), and competent cells were harvested at midlog phase culture (OD_600_ = 0.391). After electroporation (12.5 kV/cm, 20 ms), the samples were immediately plated using two plating methods: direct plating or overlay cultivation. The transformation efficiency for direct plating was 7.5 × 10^2^ ± 2.2 × 10^2^ colonies/*μ*g DNA, while that of overlay cultivation was 2.7 × 10^2^ ± 4.0 × 10 colonies/*μ*g DNA. Thus, the transformation efficiency for direct plating was 2.7-fold higher than that of overlay cultivation. The experiments were repeated in triplicate.

To analyze the effect of DNA topology on homologous recombination via double-crossover events, circular and linear marker cassettes containing 800 bp homologous regions were tested (Figure 1, i). The experimental conditions were identical to those described in the previous paragraph, except that 300 ng of circular DNA pyrElacS800 (placSpyrE) and another previously reported electric parameter (15 kV/cm, 9 ms) [17] were utilized. When DNA was electroporated, the transformation efficiency using linear DNA was 24-fold higher than that of the circular DNA: 1.5 × 10^3^ ± 4.2 × 10^2^ colonies/*μ*g DNA and 6.2 × 10 ± 7.0 colonies/*μ*g DNA, respectively. The experiments were repeated in triplicate.

Electroporation in the presence of Ca^2+^ enhanced the transformation efficiency of *E. coli* [14]; however, this effect has not been reported in the hyperthermophilic genus *Sulfolobus*. For validation of the effect of CaCl_2_ treatment on homologous recombination efficiency in *S. acidocaldarius*, electrotransformation was performed in the presence and absence of CaCl_2_ (Figure 1, ii). Competent cells were collected at midlog phase (OD_600_ = 0.420). Concentrations of 0.1, 0.2, and 0.4 mM CaCl_2_ were selected because these concentrations did not cause arching during electroporation. However, CaCl_2_ treatment did not improve transformation efficiency when compared with control experiments (data not shown). The experiments were repeated in triplicate. We speculated that DNA volume is important for improving transformation efficiency with CaCl_2_ treatment (Figure 1, iv). However, DNA volume (1000 ng) did not improve the transformation efficiency with CaCl_2_ treatment (data not shown).

We confirmed the effects of various regeneration conditions on transformation efficiency after electroporation (Figures 1, vi and 2) because our previous transformation protocol [8] did not conduct regeneration. The highest number of transformants was obtained with MBS buffer when compared with the control (without regeneration). The transformation efficiency was approximately 13-fold higher than that of the control (2.8 × 10^4^ ± 6.9 × 10^3^ colonies/*μ*g DNA and 2.3 × 10^3^ colonies/*μ*g DNA, resp.). The experiments were repeated in triplicate.

Homologous recombination efficiencies for cells harvested at different phases of cell growth (early log [OD_600_ = 0.174], midlog [OD_600_ = 0.420], and stationary phase [*D*_600_ = 0.885]) were investigated (Figure 1, iii). Competent cells were transformed with 200 ng of pyrElacS800 by electroporation. Next, 20% suspensions were plated and cultivated. The transformation efficiency of fresh cultures was 2.6–4.5-fold higher than that of older cultures (midlog and stationary phases, resp.). The transformation efficiencies of early log, midlog, and stationary phases were 7.7 × 10^2^ ± 2.9 × 10^2^ colonies/*μ*g DNA, 2.9 × 10^2^ ± 2.0 × 10^2^ colonies/*μ*g DNA, and 1.7 × 10^2^ ± 3.8 × 10 colonies/*μ*g DNA, respectively. The experiments were repeated in triplicate.

Subsequently, to study the transformation efficiency using linear DNA for homologous recombination in *S. acidocaldarius* with double-crossover events, marker cassettes containing 50–10 bp 5′ and 3′ homologous regions of the target locus at both ends of the *pyrE*-*lacS* marker were constructed (Figure 1, v). Competent cells harvested at midlog phase (OD_600_ = 0.420) were transformed with 1 *μ*g of marker cassettes. The transformation efficiency increased with the length of the homologous arms (Figure 3). When DNA with 10–20 bp of flanking regions was used, no transformants grew. Transformation efficiencies slightly improved by regeneration with MBS buffer. A few colonies transformed with DNA attached to 20 bp flanking arms were detected after regeneration. Thus, efficient marker replacement was possible with as few as 30–50 bp of flanking homology of the target region.

The following set of conditions was established as the optimized transformation protocol: DNA was introduced into competent cells collected from the early log phase by electroporation. The pulse duration was 9 ms and the field strength was 15 kV/cm. After electroporation, cells were regenerated in MBS recovery buffer and the pellet was spread on plates.

### 3.2. Establishment of the MONSTER

The multiple gene knockout system with one-step PCR (MONSTER) was developed by combining a MID strategy [3] and one-step inactivation using a linear PCR product [1] (Figures 4 and 5). Two 48 bp homologous arms were used for double-crossover events (marker integration), followed by pop-out recombination at 30 bp duplicated arms for the excision of a marker cassette (unmarked gene deletion). Thus, two MONSTER primers need to be designed for incorporation of 5′, 3′, and Tg (target gene) arms into PCR products as 5′ extensions of primers (Table 2). Sequences of forward and reverse MONSTER primers that anneal with *pyrE*-*lacS* marker genes are identical although the attached flanking regions of target genes (5′, 3′, and Tg) are different. Next, the MONSTER cassette was amplified by one-step PCR using MONSTER primers. Then, we designed different constructs of MONSTER cassettes (MONSTER-phr and MONSTER-argD) for confirming the reliability (Figures 4(a) and 5(a)). The dual marker (*pyrE*-*lacS*) was utilized for effective selection of correct transformants (Figures 4 and 5).

### 3.3. Construction of a DNA Photolyase-Deficient Strain via the MONSTER

To validate the MONSTER, we constructed a mutant with an in-frame deletion of DNA photolyase. DNA photolyase-encoding gene (Saci_1227) (named *phr* in this study) has been identified as a functional gene of photoreactivation (repair of UV-damaged DNA under light conditions) [1, 18]. To disrupt *phr*, MONSTER-phr was constructed by one-step PCR (Figure 4(a)). When 1.6 *μ*g of MONSTER-phr was electroporated into SK-1 using the optimized transformation protocol with competent cells harvested at midlog phase (OD_600_ = 0.420), approximately 60 colonies grew on XT plates (Figure 4(b)). Next, three blue colonies were selected after applying 1 *μ*L of X-gal solution (10 mg/mL) onto the plates for 1 h at 75°C (Figure 4(b)). Two blue colonies were purified by single isolation and analyzed by PCR screening using primers phr-out-F/R (Figure 4(a)). As shown in Figure 4(d), the two colonies were positive intermediate transformants (named DP-1 Int-1 and Int-2). A total of 2.3 × 10^8^ DP-1 Int cells were spread on XTU plates containing 5-FOA for pop-out recombination. X-gal visualization of the plates indicated that blue and white colonies formed with a ratio of 100 : 13 (Figure 4(c), 65 ± 35 white colonies grew). Ten 5-FOA^r^ white colonies were randomly selected for PCR analysis. The genotypes of 9 out of ten colonies were expected with an approximate 1.3 kb deletion in the *phr* locus (Figure 4(d)). One correct Δ*phr* in-frame mutant confirmed by sequencing was designated as *S. acidocaldarius* strain DP-1 and used for phenotypic analysis.

### 3.4. Characterization of the DNA Photolyase-Deficient Strain DP-1

To characterize the DNA photolyase-deficient strain, the growth properties of wild-type (SK-1) and Δ*phr* (DP-1) under light or dark conditions after UV irradiation were investigated (Figure 6). When both strains were not irradiated with UV light, their growth properties were identical under light and dark conditions. In addition, the growth of UV-treated DP-1 under dark conditions was similar to that of the host strain. In contrast, the UV-exposed DNA photolyase-deficient strain DP-1 grew slower when compared with the SK-1 strain under light conditions, indicating that deletion of the *phr* locus eliminated photoreactivation.

### 3.5. Construction of the *argD*-Deficient *S. acidocaldarius* Strain SK-5 via the MONSTER

We disrupted the *argD* gene using the MONSTER to establish a robust unmarked gene disruption system, and a positive selectable marker in *S. acidocaldarius* (Figure 5). *argD* (Saci_1363) encodes arginine decarboxylase, which catalyzes _L_-arginine to produce agmatine [19], and is a homolog to SSO0536 in *S. solfataricus* P2 and *Sis*M164_1585 in *S. islandicus* M.16.4, sharing 73% and 74% identity by Blastp analysis, respectively. For construction of the *argD* in-frame deletion mutant (Figure 5(a)), 2 *μ*g of one-step constructed MONSTER-argD was introduced into SK-1 cells harvested at the late-log phase (OD_600_ = 0.558; electroporation conditions: 12.5 kV/cm and 20 ms) and then cultivated on XT plates containing 200 *μ*g/mL agmatine at 75°C for 6 days. As shown in Figure 5(b), five colonies grew. X-gal selection revealed three blue colonies. Two of these blue colonies were purified on XT plates and analyzed by PCR screening using primers argD-F-F/R (Figure 5(a)). As shown in Figure 5(d), both clones contained 2.5 kb (*pyrE*-*lacS* marker and 30 bp 5′ regions) inserted bands, indicating that two blue colonies were positive intermediate transformants (named SK-5 Int-1 and Int-2). These transformants grew in XT liquid culture, suggesting that insertion of the marker between the stop codon and the 3′ region of the *argD* locus did not affect arginine decarboxylase activity (data not shown). A total of 3.4 × 10^8^ SK-5 Int cells were spread on XTU plates containing 5-FOA and 1 mg/mL agmatine for pop-out recombination. X-gal visualization demonstrated that blue and white colonies formed with a ratio of 167 : 16 (Figure 5(c), 16 ± 6 white colonies grew). Twelve 5-FOA^r^ white colonies were randomly selected for PCR analysis using outer primers. The genotypes of 10 out of twelve colonies showed the expected approximately 0.4 kb deletion in the *argD* locus (Figure 5(d)). One correct Δ*argD* in-frame deletion mutant confirmed by sequencing, designated *S. acidocaldarius* strain SK-5, was characterized for phenotypic analysis.

### 3.6. Characterization of the *argD* Deletion Mutant SK-5

The growth of the *argD*-deficient strain SK-5 (Δ*pyrE* Δ*suaI* Δ*argD*) was studied using XTU liquid culture in the presence or absence of agmatine (Figure 7). When SK-5 was cultivated in the presence of 1 mg/mL agmatine, growth was slightly retarded when compared with that of the host strain in the absence of agmatine. Particularly, the slowed growth of SK-5 became more striking at lower concentrations of agmatine. In contrast, SK-5 was not grown with less than 100 *μ*g/mL agmatine.

### 3.7. Construction of a Stringent-Positive Selection Marker System Based on Agmatine Selection in *S. acidocaldarius*

The agmatine selection system has been reported as a stringent-positive selection marker system in the hyperthermophilic archaea *Pyrococcus furiosus* and *S. islandicus* [10, 20]; however, this system has not been developed in *S. acidocaldarius*. To establish a selection marker system based on complementation of the *argD* gene, a *S. acidocaldarius–E. coli* shuttle vector pSAV2-argD was constructed by replacing the *S. solfataricus pyrEF* marker genes of pSAV2 with the *S. solfataricus argD* gene (SSO0536) (Figure 8(a)).

Host strain SK-5 (Δ*pyrE* Δ*suaI* Δ*argD*) cells harvested at early to midlog phase (OD_600_ = 0.308) were transformed with 8 ng of plasmid DNA (1 *μ*L) by electroporation (15 kV/cm, 9 ms) and spread on XTU plates (regeneration in recovery buffer was not conducted). Next, a previously published host–vector system based on complementation of the *pyrE* gene, SK-1 (Δ*pyrE* Δ*suaI*), and plasmid vector pSAV2 [8] was analyzed under the same transformation conditions, except that competent cells were harvested at midlog phase (OD_600_ = 0.420) and transformants were cultivated on XT plates. When SK-5 was transformed with pSAV2-argD, approximately 1.3 × 10^2^ ± 3.8 × 10 colonies grew with a transformation efficiency of 1.6 × 10^4^ ± 4.7 × 10^3^ colonies/*μ*g DNA (Figure 8(b)). This result was similar (slightly lower) to the transformation efficiency of SK-1. Approximately 3.2 × 10^2^ ± 9.8 × 10 colonies grew with a transformation efficiency of 4.0 × 10^4^ ± 1.2 × 10^3^ colonies/*μ*g DNA. In addition, no colonies were formed in the control experiments with either selection system (without electroporation and plasmid vector) (Figure 8(b)).

## 4. Discussion

The goal of the present study was to establish a multiple gene knockout system with PCR tailing in the thermoacidophilic crenarchaeon *S. acidocaldarius*. For this, we first optimized the transformation protocol by characterizing the effects of transformation conditions on transformation efficiency. Next, we successfully developed a multiple gene knockout system with one-step PCR (MONSTER) by combining marker recycling with PCR tailing. This technique allows for the simple one-step construction of an unmarked gene knockout cassette and isolation of targeted gene deletion mutants. Unmarked gene deletion methodologies have been troublesome for genetic studies of other recombinogenic hyperthermophilic archaea. Although the development of PCR-tailing methods is possible for hyperthermophilic archaea, the potential for multiple gene knockout systems is limited due to the limited selectable marker systems. Thus, the MONSTER may be a speedy and powerful genetic tool for other recombinogenic hyperthermophilic archaea. In addition, we also constructed a stringent selectable marker system using agmatine, which provides the basis for further genetic manipulation in *S. acidocaldarius.*

Our results indicated that the main factors affecting transformation (homologous recombination) efficiency via double-crossover events were DNA topology, recovery conditions after electroporation, and flanking region length. In addition, the plating methods and the growth phase of competent cells were also important for optimizing transformation. In contrast, CaCl_2_ treatment and DNA volume did not affect transformation efficiency in this study.

The effects of DNA form on homologous recombination were reported in *Sulfolobus* species [2, 13]. Our results support a previous report that the transformation efficiency using linear DNA was higher than that of circular DNA [2, 13].

To develop a gene manipulation system based on PCR tailing, we focused on the possibility of sufficient homologous recombination via double-crossover events with very short homologous regions. The effects of flanking region length on homologous recombination efficiency in *S. acidocaldarius* were previously reported by Kurosawa and Grogan [13], and our data support their findings (Figure 3). The PCR-tailing technique was also previously established [1]. In contrast, our study is the first to report that sufficient transformation efficiency for gene manipulation was demonstrated even with very short (30–50 bp) flanking homologous arms. When 40 bp homologous arms were attached, the transformation efficiency using our protocol (20 ± 7 colonies/*μ*g) was slightly higher than that of the recombinogenic *P. furiosus* strain COM1 (6 colonies/*μ*g) reported by Farkas et al. [21] (Figure 3). To our knowledge, no similar observation has been reported in the literature.

**Figure 2:**
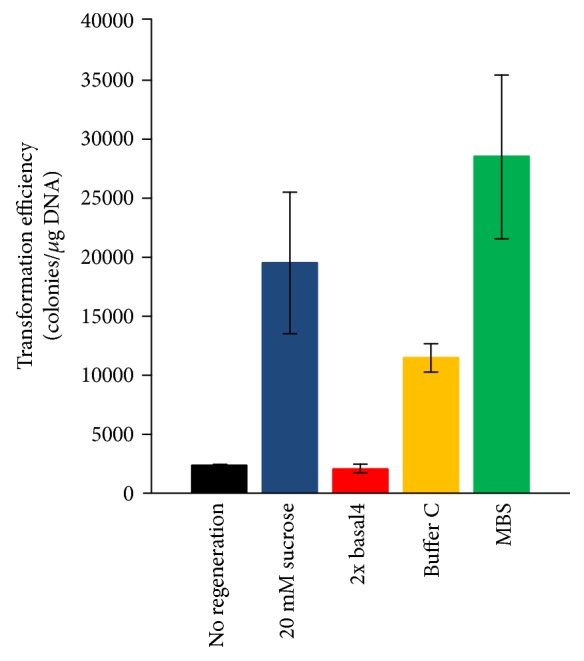
Effects of recovery conditions on transformation efficiency. SK-1 cells harvested at early to midlog phase (OD_600_ = 0.308) were transformed with 200 ng of pyrElacS800. After electroporation (15 kV/cm, 9 ms), the cell suspension was transferred to 800 *μ*L of recovery buffer (20 mM sucrose, 2× basal4, Buffer C, and MBS) and incubated. Ten percent of the regenerated sample was mixed with 10 mL of top gel solution and poured onto XT plates by overlay cultivation. Error bars represent the standard deviation of three independent experiments.

**Figure 3:**
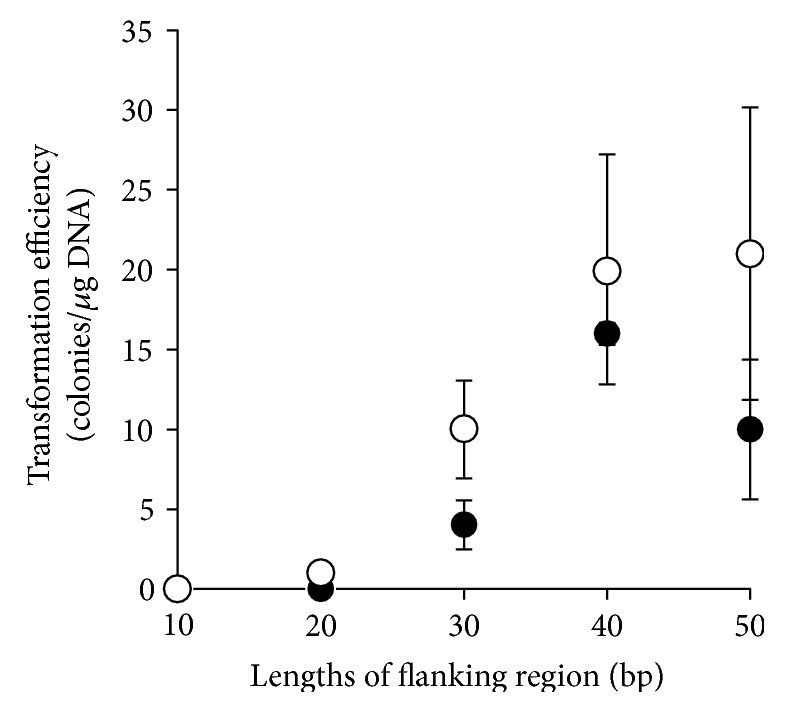
Effects of length of the flanking homologous region on transformation efficiency. SK-1 was transformed with 1 *μ*g of a linear marker cassette attached to flanking regions (10–50 bp) of various lengths at both ends. The resulting colonies were counted. Open circles: regeneration in MBS; closed circles: direct plating (no regeneration). Error bars represent the standard deviation of three independent experiments.

Homologous recombination (via double-crossover events) efficiencies using linear DNA have been reported in three hyperthermophilic archaea: *Thermococcus kodakarensis* KOD1, 10^2^ colonies/*μ*g linear DNA containing 1 kb flanking regions [22]; *P. furiosus* COM1 (parent strain DSM 3638), 2.9 × 10^3^ colonies/*μ*g linear DNA containing 1 kb flanking regions [21]; *S. islandicus* M.16.4, 20–30 colonies/*μ*g linearized DNA (pC-SsoargD) containing 755 and 671 bp flanking regions [10] and 10–50 colonies/*μ*g linearized DNA (pMID-apt) containing 703 and 617 bp flanking regions [23]; and *S. islandicus* REY15A, 10–200 colonies/*μ*g linearized DNA (pKL2) containing 1.5 kb flanking regions [2]. The homologous recombination efficiency reported in our current study (10^2^-10^3^ colonies/*μ*g DNA) was higher than that of *T. kodakarensis* and *S. islandicus* and nearly identical to that of *P. furiosus*. However, these are not direct comparisons because the experimental conditions were different (e.g., size of the flanking regions and type of DNA construct). Notably, when transformed cells were regenerated under MBS buffer (Figure 2), the transformation efficiency (2.8 × 10^4^ ± 6.9 × 10^3^ colonies/*μ*g DNA) was similar to that of *S. acidocaldarius* transformed with plasmid vector (1.6 × 10^4^ ± 4.7 × 10^3^ colonies/*μ*g pSAV2-argD and 4.0 × 10^4^ ± 1.2 × 10^3^ colonies/*μ*g pSAV2) (Figure 8(b)). This high transformation efficiency will facilitate genetic studies and provide powerful advantages for the development of further genetic tools in this archaeon [24–26].

Improvement of electrotransformation efficiency by CaCl_2_ treatment in *S. acidocaldarius* was previously described (S. Suzuki and N. Kurosawa, presented at the Bioscience, Biotechnology, and Agrochemistry Convention, Japan, 27–30 March 2016); however, our study did not confirm this finding. Thus, further study is necessary to address this discrepancy.

**Figure 4:**
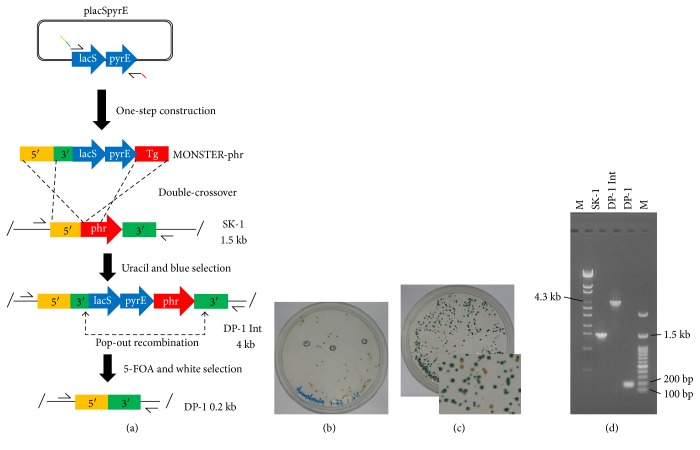
Schematic of the multiple gene knockout system with one-step PCR (MONSTER). (a) Construction of a DNA photolyase-encoding gene (*phr*) deletion mutant. A plasmid-borne *pyrE*-*lacS* marker served as the PCR template, which attached *S. acidocaldarius* chromosomal sequences (5′, 3′, and partial sequences of *phr* at the 5′ ends of the primers) to the ends of the selectable dual marker. After one-step construction, the MONSTER-phr was electroporated into strain SK-1. A double crossover between the MONSTER-phr and the chromosome at the 5′ and Tg regions results in the *pyrE*-*lacS* marker and 3′ region insertion at the *phr* locus. The resulting uracil prototroph transformants that exhibit blue colonies can be selected on uracil-free plates. A DNA photolyase deletion mutant with the marker removed was generated by pop-out recombination at two duplicated 3′ regions, which can be selected by 5-FOA counterselection in combination with X-gal staining. Arrows show the positions of PCR primer sets. (b) Uracil and blue selection plate. (c) 5-FOA and white selection plate. (d) PCR analysis of the *phr* locus of the *S. acidocaldarius* strains SK-1 (Δ*pyrE* Δ*suaI*), DP-1 Int (intermediate), and DP-1 (Δ*pyrE* Δ*suaI* Δ*phr*) using phr-out-F/R as primers. The expected sizes of the PCR bands were 1.5 kb (wt), 4 kb (recombinant), and 0.2 kb (deletion mutant). A *λ*-EcoT14 or 100 bp DNA ladder was loaded in lane M.

**Figure 5:**
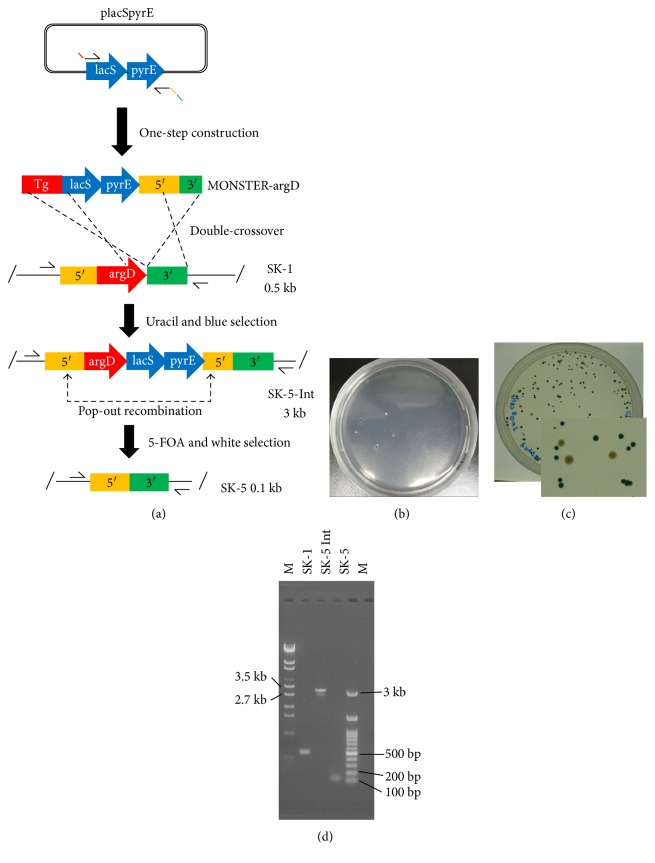
In-frame deletion of *argD* via the MONSTER. (a) Construction of an *argD* deletion mutant. A plasmid-borne *pyrE*-*lacS* marker served as the PCR template, which attached *S. acidocaldarius* chromosomal sequences (5′, 3′, and partial sequences of *argD* at the 5′ ends of the primers) to the ends of the selectable dual marker. After one-step construction, the MONSTER-argD was electroporated into strain SK-1. A double crossover between the MONSTER-argD and the chromosome at the Tg and 3′ regions results in the *pyrE*-*lacS* marker and 5′ region insertion at the *argD* locus. The resulting uracil prototroph transformants exhibit blue colonies and can be selected on uracil-free plates. An *argD* deletion mutant with the marker removed was generated by pop-out recombination at two duplicated 5′ regions, which can be selected by 5-FOA counterselection in combination with X-gal staining. Arrows show the positions of PCR primer sets. (b) Uracil and blue selection plate. (c) 5-FOA and white selection plate. (d) PCR analysis of the *argD* locus of the *S. acidocaldarius* strains SK-1 (Δ*pyrE* Δ*suaI*), SK-5 Int (intermediate), and SK-5 (Δ*pyrE* Δ*suaI* Δ*argD*) using argD-F-F/R as primers. The expected sizes of the PCR bands were 0.5 kb (wt), 3 kb (recombinant), and 0.1 kb (deletion mutant). A *λ*-EcoT14 or 100 bp DNA ladder was loaded in lane M.

**Figure 6:**
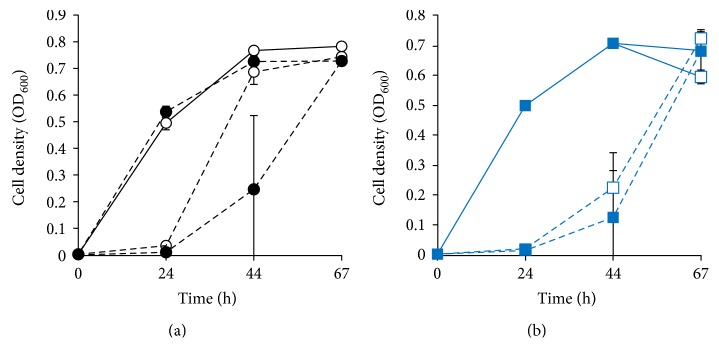
Growth curve of DNA photolyase-deficient strain after UV irradiation. Wt (SK-1) and Δ*phr* (DP-1) cells were irradiated with UV light (1200 J/m^2^) and cultivated in XTU liquid medium under light or dark conditions for viability testing. (a) SK-1. (b) DP-1. Open symbols: light conditions; closed symbols: dark conditions; solid lines, without UV irradiation; dotted lines: with UV irradiation. Error bars represent the standard deviation of two independent experiments.

**Figure 7:**
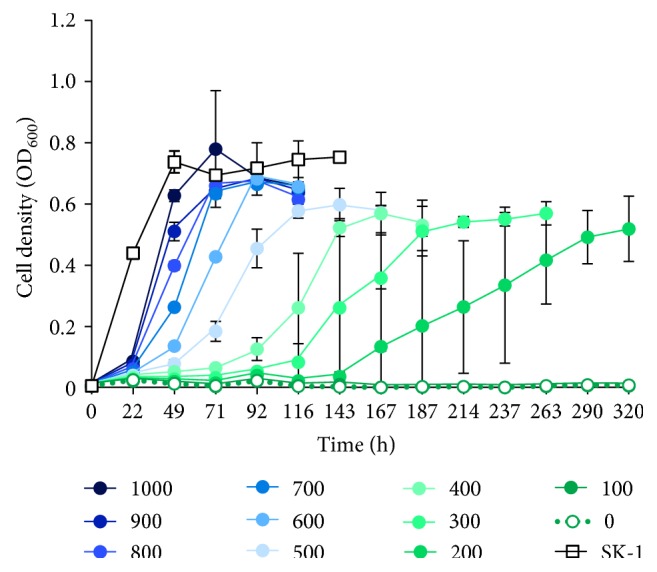
Growth curve of the *argD* deletion mutant SK-5. Wt (SK-1) and Δ*argD* (SK-5) were cultivated in XTU liquid medium with or without agmatine at 75°C with shaking. Closed circles: SK-5 with agmatine (100–1000 *μ*g/mL); open circles: SK-5 without agmatine; open squares: SK-1 without agmatine. Error bars represent the standard deviation from three independent experiments.

Effective multiple gene knockout techniques have been developed in *Sulfolobus* [2–4]. However, the cloning steps of two to four fragments for construction of knockout vectors are required for these genetic tools. In addition, the screening of positive clones that contain the correct construct must be randomly selected during subcloning because X-gal selection cannot be utilized. In contrast, PCR tailing is a high-throughput gene knockout technique [27]. However, the possibility of using this method for multiple gene knockout is limited in *S. acidocaldarius* because marker genes are lacking [1]. We developed the MONSTER by combining the multiple gene knockout technique with PCR tailing in *S. acidocaldarius*. The main advantage of the MONSTER compared with published unmarked gene deletion methodologies [2–4] is the very simple construction of multiple gene knockout cassettes without any plasmid construction. The usefulness of this technique was proven by unmarked gene knockout of the *phr* and *argD* genes. Another advantage of the MONSTER is that multiple unmarked gene knockout cassettes can also be simultaneously amplified under the same PCR conditions because the sequences of MONSTER primers that anneal with the dual (*pyrE*-*lacS*) marker genes are identical, even though the attached flanking regions of the target genes are different. Therefore, MONSTER is a high-throughput method compared with the widely used methods in *Sulfolobus* [2–4]. Notably, the purification of intermediate transformants (Int strain) was very important for pop-out selection (Figures 4(c) and 5(c)). Thus, this study provides an alternative and versatile strategy for the genetic manipulation of *S. acidocaldarius* with several advantages.

To establish the MONSTER in other hyperthermophilic archaea, dual marker genes are required for counterselection and screening (Figures 4(c) and 5(c)). In addition, recombinogenic host strains that allow for homologous recombination using very short flanking homologous regions is likely required.

**Figure 8:**
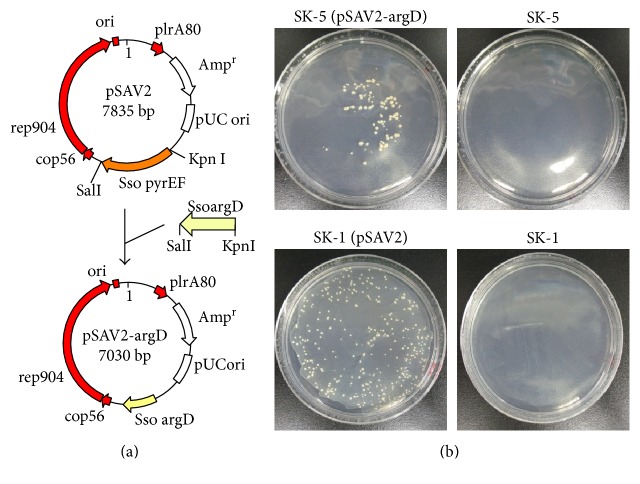
Development of a transformation system based on agmatine selection for *S. acidocaldarius*. (a) Construction of pSAV2-argD. The *S. solfataricus pyrEF* operon was replaced by a 0.6 kb *S. solfataricus argD* marker. The resulting vector was named pSAV2-argD. (b) Transformation of the *argD* deletion mutant SK-5 with pSAV2-argD. Plating of SK-5 (Δ*pyrE* Δ*suaI* Δ*argD*) transformed with 8 ng of plasmid DNA of pSAV2-argD (SK-5 [pSAV2-argD]) on an XTU plate at 75°C for 10 days and SK-1 (Δ*pyrE* Δ*suaI*) transformed with 8 ng of plasmid DNA of pSAV2 (SK-1 [pSAV2]) on an XT plate at 75°C for 7 days are shown. Controls (SK-1 and SK-5) included competent cells without plasmid DNA and electroporation.

A uracil-based selection system (*pyrE*-, *pyrF*-, or *pyrEF*-deficient strains and marker genes) cannot efficiently estimate transformation efficiency in hyperthermophilic archaea due to the interference caused by background growth of the *pyrEF*-deficient strain on solid medium [10]. In contrast, an agmatine selection system is a powerful genetic marker due to the lack of background colony growth on plates (Figure 8(b)) [10]. Therefore, the genetic marker system developed in this study will allow versatile genetic manipulation in *S. acidocaldarius*. Notably, a higher concentration of agmatine was required for cultivation of the *S. acidocaldarius argD*-deficient strain when compared with other hyperthermophiles [10, 20, 23, 28].

We previously reported that no background colonies appeared in the host–vector system (especially the SK-1 strain) using uracil selection for a 7-day cultivation [8]. This advantage was confirmed with our stringent positive marker system based on agmatine selection (Figure 8(b)).

Additionally, we constructed the DNA photolyase-deficient strain DP-1 as a genetic host strain that does not require dark conditions for the functional genetic analysis of candidate genes involved in the UV response [29–31].

## 5. Conclusion

We combined marker recycling (pop-out recombination) with PCR tailing to develop a multiple gene knockout system with one-step PCR. In addition to the widely used multiple gene knockout techniques in *S. acidocaldarius*, this study describes an alternative strategy that enables the very simple construction of multiple gene knockout cassettes. Indeed, we believe our techniques will contribute to the genetic study of this archaeon.

## Figures and Tables

**Table 1:** Strains and DNA used in this study.

Strain or plasmids	Relevant characteristic(s)	Source or reference
Strains		
*S. acidocaldarius*		
SK-1	MR31 [32] with Δ*suaI*	[8]
DP-1 Int	SK-1 with Δ*phr*::3′ region of *phr-pyrE-lacS*	This study
DP-1	SK-1 with Δ*phr*	This study
SK-5 Int	SK-1 with Δ*argD*::5′ region of *argD-pyrE-lacS*	This study
SK-5	SK-1 with Δ*argD*	This study
Plasmids		
pSAV2	*Sulfolobus-E. coli* shuttle vector, based on pBluescript II KS (−) and pRN1, with the *SsopyrEF* marker	[8]
pSAV2-argD	*SsopyrEF* marker in pSAV2 replaced by *SsoargD* marker	This study
pSuaIPOP	pBluescript II KS (−) carrying the 800 bp of 5′ and 3′ regions of *suaI*, *pyrE*, and 800 bp of 3′ region of *suaI*	[8]
placSpyrE	pSuaIPOP derivative carrying 800 bp of 5′ and 3′ homologous regions of *suaI* locus at both ends of *pyrE*-*lacS* dual marker	This study
PCR product		
pyrElacS800	Linear DNA carrying 800 bp of 5′ and 3′ homologous regions of *suaI* locus at both ends of *pyrE*-*lacS* dual marker	This study

**Table 2:** Primers used in this study.

Primer	Sequence^a^ (5′-3′)
SSOlacS-F	TTTCTGCAGTGTTTTTCTCTATATCAATCTC
SSOlacS-R	TTTGGATCCATCTAAATGACTTTCCAATTAG
E800-F	ACTTCTCCTCCTTATTATAACG
E800-R	GGATTCTCTTACTTTTCTAAAG
E50-F	TGAGGGAAAAAATAAACGAAAAG
E50-R	GACCTTGAATTTGAAGTGGC
E40-F	AATAAACGAAAAGTTAGAAAAGAA
E40-R	TTGAAGTGGCGTCTCTAGAT
E30-F	AAGTTAGAAAAGAAATCTCAG
E30-R	GTCTCTAGATCGTTAGCAC
E20-F	AGAAATCTCAGTGACTGCAG
E20-R	CGTTAGCACATAAAGTCAGTC
E10-2F	ATCTCAGTGATGTTTTTCTCTATATCAATCTC
E10-2R	TAAAGTCAGTACTCCTAGATCTAAAACTAAAG
phr-pop-F	ATGATGTTTTAGAACAAAAGATTATTTAGTTGTAGTATAATCATTAGTCCAAGGGGAAAAAGTATAAAGAGAAAACAA**TGTTTTTCTCTATATCAATCTC**
phr-pop-R	ATTATCGAATAATCTCAAATCCCTTCTAAATATTACTGCACAATCCAC**ACTCCTAGATCTAAAACTAAAG**
phr-out-F	AACGCTGGGATGCTGATAAG
phr-out-R	ATGACCAGACTACTAACGTAC
argD-pop-F	AAACCTAAACGTCATCAAATGTTTTTCGCAGATAGAAGTTCAGAGTGA**TGTTTTTCTCTATATCAATCTC**
argD-pop-R	AATAGGTAGATGATGAAATTAAAAAAAGAGATCGATGATCAACTCAGCCTCCTTTTCTATTACCCTCCATCACCACTT**ACTCCTAGATCTAAAACTAAAG**
argD-F-F	TTACTTTATATATCTCATTCTG
argD-F-R	CTAATTAGGGAAATTGGTTAC
SsoargD-KpnI-F	TTTGGTACCCTTATTACCTAGATATAACGTT
SsoargD-PstI-R	TTTGTCGACTACTGCTTTGATCAAATATAAG

^a^Restriction sites are underlined and sequences of MONSTER primers that anneal with the *pyrE*-*lacS* marker genes are in bold.
